# Editorial: Machine learning for biological sequence analysis

**DOI:** 10.3389/fgene.2023.1150688

**Published:** 2023-02-03

**Authors:** Zhibin Lv, Mingxin Li, Yansu Wang, Quan Zou

**Affiliations:** ^1^ College of Biomedical Engineering, Sichuan University, Chengdu, China; ^2^ Institute of Fundamental and Frontier Sciences, University of Electronic Science and Technology of China, Chengdu, China; ^3^ Yangtze Delta Region Institute (Quzhou), University of Electronic Science and Technology of China, Quzhou, China

**Keywords:** machine learning (ML), biosequence analysis, biomarker, BERT classification algorithm, single-cell, imblanced data

## 1 Introduction

Biomacromolecules, primarily proteins, DNA, and RNA, are crucial for vital physiological processes. Biomacromolecules can generally be represented by sequences, comprising series of strings, which are referred to as bio-sequences and represent the primary structures of proteins, DNA, and RNA. The development of sequencing technologies, particularly next-generation sequencing and tandem mass spectrometry, has led to the production of vast amounts of bio-sequence data. It is established that structure generally determines function; in particular, determination of tertiary structure is critical for functional analysis of biomacromolecules. Nevertheless, determination of the tertiary structure of bio-sequences is relatively difficult; therefore, directly obtaining functional information from primary structures (i.e., bio-sequences) is extremely challenging and an important problem that requires urgent resolution.

Machine learning technologies, based on statistical theory and data mining, provide new tools for bio-sequence analysis that are effective for biological function analysis of genes and proteins, as well as determining relationships between primary structure and function. There are two basic problems in the use of machine learning in this context that have yet to be satisfactorily resolved. One is how to extract sufficient and effective features from bio-sequences. Usually, bio-sequences comprise series of strings that must be converted into numerical vectors before input into machine learning models, a process referred to as feature extraction. Only by effectively extracting the hidden numerical features in primary structure sequences can they be successfully mined by the machine learning model and achieve optimal function recognition. The other problem is that of data imbalance, which refers to the fact that the ratio of positive to negative sample sequences are not 1 to 1; in actual application, there are generally fewer positive than negative samples. To obtain the best results, machine learning models often need to be trained with balanced data, and unbalanced data will greatly affect training of machine learning models and their application in real-world scenarios. At present, some methods have been proposed to solve the problem of data imbalance, but they still cannot satisfactorily solve this fundamental issue. In this Research Topic, we focus on the two challenges described above, as well as collating the results of recent research on related Research Topic. The total of 12 articles can be divided into three categories, as follows: 9 papers on the identification of functions and interactions based on bio-sequences, 1 paper on a bioinformatics tool recommendation platform, and 2 papers on biomarker mining and analysis.

## 2 Identification of functions and interactions of bio-sequences

Identification of the biological functions of macromolecular sequences directly from their primary structures has been a hotspot in the application of machine learning. We collected 9 papers related to this Research Topic, which explore a variety of feature extraction techniques and machine learning methods for different bio-sequences, and achieved the most advanced accuracy in corresponding function identification.

Sucrose transporter (SUT) is a transmembrane protein that occurs widely in plant species and has important roles in sucrose transport and sucrose-specific signal transduction. Chen et al. built a model named ISTRF, based on a random forest algorithm, to identify SUT proteins by constructing an in-house dataset comprising SUT and non-SUT sequences, then using feature extraction tools including: protein amino acid composition, transition, and distribution; position-specific scoring matrix (PSSM) composition; and k-separated-bigrams-PSSM. They also applied the Borderline-SMOTE algorithm to solve the problem of data imbalance. ISTRF achieved an independent test accuracy of 96.1%.

Moonlighting proteins are present in many animals, plants, and microorganisms and play important roles in signal transduction, cell growth and motility, tumor suppression, DNA synthesis and repair, and metabolism of biological macromolecules. Chen et al. used linear discriminant analysis (LDA) and a support vector machine ensemble with bagging (bagging-SVM) to build a bioinformatics tool that can effectively identify moonlighting proteins. The tool uses three embedded features to encode proteins, a linear discriminant method for feature selection, and a SVM as the classifier. The authors found that the LDA method can effectively screen out sequence features identifying moonlight proteins, and that the bagging-SVM is superior to a classic SVM algorithm, achieving accuracy of 93.25%.

As a good substitute for antibiotics, antimicrobial peptides (AMPs) can effectively kill bacteria in organisms, resulting numerous therapeutic effects, such as antibacterial, wound healing, antioxidant, and immune regulation activities. Dong et al. proposed a deep learning model that fuses multiple sequence feature representations (four types) as input to identify AMPs. They adopted a convolutional layer structure and fully connected layers to construct a deep learning network; model accuracy was 97.8%.

Vesicle transporters are membrane proteins that function by regulating the interaction of specific molecules with vesicle membranes. Fan et al.established a hypergraph regularized K-local hyperplane distance nearest neighbor machine learning model to distinguish vesicle transporters from non-vesicle transporters [4]. The sequence encoding feature used by this model is PsePSSM. The research showed that the classifier outperformed traditional classifiers and achieved an accuracy of approximately 84%.

Protein-protein interactions (PPIs) are fundamental to deep understanding of proteome functional mechanisms and are highly valuable in medical applications of novel diagnostic and therapeutic targets. Yang et al. developed a new tool for PPI data and functional analysis [5]. A key feature of the tool is that each protein involved in a PPI is encoded using Gene Ontology and Kyoto Encyclopedia of Genes and Genomes (KEGG) pathway annotations. The tool uses minimum absolute shrinkage and selection operators, gradient boosting machines, maximum correlation, and minimum redundancy to rank features for importance analysis. Then, the most significant features were selected as critical functional items identified by PPI.

Pseudouridine is an abundant RNA modification that can affect RNA stability and immunoreducibility, among other characteristics, and its mutation is associated with numerous malignancies, including lung and gastric cancers. Zhang et al.proposed a new machine learning model, PseU-ST, to identify RNA pseudo-uridine modification sites in *Homo sapiens*, *Saccharomyces cerevisiae*, and *Mus musculus*. They used six feature extraction methods to encode RNA, and chi-square analysis for feature selection. In addition, stacking ensemble learning was applied. The accuracy values of PseU-ST for data from *H. sapiens*, *S. cerevisiae*, and *M. musculus* were approximately 94%, 88%, and 89%, respectively.

Inspired by the hypothesis that pathogen-derived immunological epitopes can mediate CD8^+^ T cell-associated host adaptive immune responses, Hu et al. used available positive and negative CD8^+^ T cell epitope (TCE) data to propose a novel predictor, CD8TCEI-EukPath, to detect CD8^+^ TCEs in eukaryotic pathogens. The authors aimed to develop a method to enable rapid screening of epitope-based vaccine candidates. CD8TCEI-EukPath integrated three hybrid features, adopted an MRMD tool for feature selection, and used a LightGBM classifier to distinguish CD8^+^ TCEs from non-CD8^+^ TCEs, thereby achieving accuracy values of approximately 79% and 78% in cross-validation and independent testing, respectively.

The success of a transformer model with a unique self-attention mechanism in natural language processing inspired Mai et al. to use it to predict and analyze promoters in *Synechococcus* sp. and *Synechocystis* sp. They named the tool, TSSNote-CyaPromBERT, and it facilitates large dataset extraction, model training, and promoter prediction from public dRNA-seq datasets. The model of TSSNote-CyaPromBERT achieved an area under the receiver operating curve value of 0.92 for distinguishing promoter and non-promoter nucleotides, as well as relatively good performance in cross-species verification testing. Monte Carlo sampling and attention score visualizations can be used to explain the model behavior.

Histone modifications affect various chromatin-dependent processes, including DNA replication, repair, and transcription. Chen et al. proposed a new deep learning model, TransferChrome, with the aim of solving the problem of inaccurate gene expression prediction across cell lines, based on use of a self-attention mechanism to predict the effects of histone modifications on gene expression, and used a transfer learning model to achieve cross-cell gene expression prediction. The authors trained and tested TransferChrome on 56 different cell lines from the REMC database, and achieved a mean area under the curve score of 84.79%.

## 3 Mining of disease-related markers

COVID-19 triggers a complex immune response, where CD8^+^ T cells play a particularly important role in controlling disease severity. The mechanisms underlying the regulatory effects of CD8^+^ T cells on COVID-19 remain poorly studied. Lu et al. applied single-cell omics data to target three CD8^+^ T cell subtypes and three COVID-19 disease states, using CD8^+^ T single cell data expression profiles, combined with multiple feature selection methods, to screen out biomarkers, including ZFP36, DUSP1, TCR, and IL7R, among other molecules. They proposed that these genes can be confirmed to play an immunomodulatory role in the processes of infection with and recovery from COVID-19 disease. Simultaneously, the authors used the characteristics of CD8^+^ T cell subtypes to establish a machine learning model that can distinguish COVID-19 disease severity.

Discovering tumor markers related to cancer has long been a focus of considerable research attention. Zhao et al. analyzed the expression level of KRAS, a signal transduction protein that binds to GTP in the MAPK pathway. To assess the tumor microenvironment, they used 22 immune-infiltrating cell expression datasets to calculate immune and stromal scores. They also used 33 tumor expression datasets to construct a PPI network by establishing KRAS, immune checkpoint, and interacting genes. By performing gene set enrichment analysis, they generated results suggesting that KRAS may be a reliable prognostic biomarker for diagnosing patients with cancer that can be incorporated into tumor-targeted drugs.

## 4 An online platform for recommendation of bioinformatics tools

How to choose a suitable tool for structural variation analysis of bio-sequence data is a particularly interesting problem. Numerous bioinformatics tools have been developed, but their applicability to real data and universality are serious concerns, and it is unrealistic to test each tool individually. Wang et al. noticed this problem and developed a meta-learning framework to establish the relationship between data features and bioinformatics tool performance. Using random forest analysis, the authors identified the relationships between 8 selected data features and the optimal caller, and used these relationship to recommend callers. Testing the algorithm of the automatic recommendation tool constructed showed that the applicable samples varied among different callers. Hence, different tools are often suitable for various types of bio-sequencing data analyses. The accuracy of recommended tools was maintained above a mean of 80%, which is far superior to random selection or fixed selection strategies. The authors also built an online website and provided the source code.

In conclusion, the papers discussed in this Research Topic demonstrate significant roles for machine learning techniques in various bio-sequence analysis applications, and we sincerely hope that this Research Topic will be widely read and benefit readers. In particular, this Research Topic collates insightful explanations and applications that can contribute to developments and advances in biology. Finally, we wish to convey our appreciation for all the efforts of the authors, reviewers, and staff of the *Frontiers in Genetics* editorial office.

